# Smart decision support system for keratoconus severity staging using corneal curvature and thinnest pachymetry indices

**DOI:** 10.1186/s40662-024-00394-1

**Published:** 2024-07-08

**Authors:** Zahra J. Muhsin, Rami Qahwaji, Mo’ath AlShawabkeh, Saif Aldeen AlRyalat, Muawyah Al Bdour, Majid Al-Taee

**Affiliations:** 1 Department of Computer Science, University of Bradford, Bradford, BD7 1DP UK; 2 Al-Taif Eye Center, Sulaiman Al Hadidi Street, Amman, Jordan; 3 School of Medicine, The University of Jordan, Amman, 11942 Jordan

**Keywords:** Corneal tomography, Keratoconus, Feature selection, Severity staging, Machine learning, Smart web

## Abstract

**Background:**

This study proposes a decision support system created in collaboration with machine learning experts and ophthalmologists for detecting keratoconus (KC) severity. The system employs an ensemble machine model and minimal corneal measurements.

**Methods:**

A clinical dataset is initially obtained from Pentacam corneal tomography imaging devices, which undergoes pre-processing and addresses imbalanced sampling through the application of an oversampling technique for minority classes. Subsequently, a combination of statistical methods, visual analysis, and expert input is employed to identify Pentacam indices most correlated with severity class labels. These selected features are then utilized to develop and validate three distinct machine learning models. The model exhibiting the most effective classification performance is integrated into a real-world web-based application and deployed on a web application server. This deployment facilitates evaluation of the proposed system, incorporating new data and considering relevant human factors related to the user experience.

**Results:**

The performance of the developed system is experimentally evaluated, and the results revealed an overall accuracy of 98.62%, precision of 98.70%, recall of 98.62%, F1-score of 98.66%, and F2-score of 98.64%. The application's deployment also demonstrated precise and smooth end-to-end functionality.

**Conclusion:**

The developed decision support system establishes a robust basis for subsequent assessment by ophthalmologists before potential deployment as a screening tool for keratoconus severity detection in a clinical setting.

**Supplementary Information:**

The online version contains supplementary material available at 10.1186/s40662-024-00394-1.

## Background

Keratoconus (KC) is a degenerative condition that affects the cornea, the transparent layer at the front of the eye. It involves the gradual central thinning of the cornea, resulting in a conical or irregular shape and causing visual impairment [1]. Both genders are not spared, and KC typically manifests in early adolescence and advances till the fourth decade of life. It asymmetrically affects both eyes and can markedly hinder vision, resulting in distorted vision, near-sightedness, and astigmatism [2]. The exact cause of KC is not fully understood despite decades of research. A mix of environmental and genetic factors is thought to influence the onset and progression of this disease [2–5].

The prevalence and incidence of KC varies in different communities around the world [6–8]. This can be attributed to the diversity of populations studied and the lack of specific guidelines for defining and classifying KC. Research indicates that, in comparison to other populations, the prevalence of KC is higher in Middle Eastern and South Asian populations. For example, the prevalence of KC in Iran has been increasing in recent years, from 1 in 126 in 2013 to 1 in 32 in 2018 [9–11]. Research studies conducted in the UK [12–19] revealed a notable variation in the prevalence of KC among individuals from different ethnic backgrounds. Most KC cases were identified in individuals of Indian descent within a community comprising 87% White and 11% Asian (comprising individuals of Indian, Pakistani, or Bangladeshi backgrounds). By examining screening data from hospital records, researchers identified 229 Asian patients and 57 White patients with KC. The researchers concluded that there was a four-fold increase in the prevalence of KC among Indians and similar Asian communities, underscoring the significant ethnic component of the disease. Most of these prevalence studies were conducted on patients in hospitals or clinics, where it was easier to gather data. This likely underestimates the disease prevalence since patients are frequently asymptomatic, making it easier to overlook the earlier and more subtle manifestations of the disease [20]. The true prevalence of KC, however, can be determined more accurately by population-based screening studies.

Management of KC is challenging because this disease can be undetectable at its early stages, and standard eyeglasses or contact lenses may allow good visual acuity. Ealy diagnosis of KC is therefore important to manage symptoms related to reduced visual acuity and astigmatism as well as to prevent disease progression. Additionally, management of KC depends on the disease's stage and involves non-surgical and surgical options [21]. Non-surgical options are usually recommended in the early stages. These include advising patients to avoid eye rubbing as well as correction of vision. Spectacles and soft contact lenses are typically used in the early stages to correct near-sightedness, far-sightedness, and astigmatism. Rigid contact lenses are used for more progressive disease stages with irregular astigmatism [22]. Although corrective glasses and lenses can correct the refractive error, they do not halt disease progression. Current practice is to proceed for corneal cross-linking for progressive or expected to progress KC cases. More advanced stages are managed surgically with a corneal ring implant or corneal transplantation (also known as keratoplasty) including partial thickness keratoplasty or full thickness (penetrating) keratoplasty for the severe conditions.

Corneal collagen cross-linking was approved by the US Food and Drug Administration (FDA) in 2016 and involves the application of a vitamin B2 (riboflavin) solution as a photosensitizer to the eye and ultraviolet light (UV-A) at a wavelength of 370 nm [23]. New collagen bonds form, restoring and preserving the cornea's strength and flat spherical shape. Clinical trials show these changes persist for up to 7 years post-initial treatment [24]. Another option is implanting a corneal ring, which involves placing a C-shaped ring inside the cornea stroma to flatten the cornea’s surface. This reduces astigmatism, which results in improved visual acuity. Corneal transplant is a highly effective surgical option in which a donor cornea replaces the patient’s damaged cornea. Studies show an excellent 5-year graft survival rate with more than 90% of patients having a corrected visual acuity of 6/12 or better [25]. However, most patients still need glasses or contact lens to provide the optimal vision after keratoplasty.

The diagnosis of KC typically relies on a combination of medical history, physical exam (including optometric refractive assessment, retinoscopy, and slit-lamp biomicroscope), and corneal imaging studies. Devices commonly used to obtain images of the cornea are corneal topography, tomography, and optical coherence tomography (OCT) [26]. Corneal topography is a special technology that maps the surface of the cornea in terms of elevation and curvature aspects of both the anterior and posterior surfaces. OCT provides high-resolution cross-sectional scans of the cornea and ocular surface. Each tool has a set of parameters that are used to provide data to aid in KC diagnosis.

In recent years, machine learning (ML), a branch of artificial intelligence, has evolved as a promising tool for aiding the identification and diagnosis of complex conditions [27, 28] including KC. Numerous supervised and unsupervised ML methods have been proposed for the diagnosis of KC. Supervised methods were trained with labelled input data to detect KC from unlabelled input data [29], while unsupervised learning used ML algorithms to identify patterns or clusters in the data [30]. Deep learning, a sub-branch of ML designed for processing large datasets [31] has also been proposed for KC detection, and is especially adept at segmenting or classifying corneal images [32]. These techniques were used to assess a wide range of parameters that were obtained from corneal imaging devices as well as other clinical and biometric variables to detect KC [33]. When given corneal topography, tomographic data, or a combination of both, many of these methods effectively distinguish between two or more classes [34].

In the context of KC severity, studies that divided KC corneas into distinct clinical stages utilizing ML algorithms were based on a range of investigations that categorised KC corneas into different stages. In the studies of Bolarín et al. [35] and Velázquez-Blázquez et al. [36], the authors graded corneas into grades I–V, employing a classification system based on corrected distance visual acuity (CDVA). In [37], the authors graded corneas as 1–4 using the Amsler-Krumeich (AK) classification system that was primarily centered on keratometry but also incorporating refraction and pachymetry [38]. Another study [39] categorized KC corneas into mild and moderate stages through a classification scheme that was self-defined. Numerous studies have presented diverse ML models to predict KC severity. However, there is no consensus on a standardized set of parameters applicable for diagnosing KC or predicting its severity [40]. This is possibly caused by the use of various diagnostic criteria, imaging instruments, and a lack of readily available datasets that can function as a reference for predicting KC severity levels [33]. Moreover, most of these studies were conducted in an academic research setting [41], rather than being applied in clinical practice [42, 43]. This challenge arises from ineffective communication between clinicians and system developers, leading to caution in relying solely on ML predictions without supplementary clinical validation.

In contrast to prior studies on KC severity classifications, this study proposes a real-world decision support system that is collaboratively developed by both ML experts and ophthalmologists. The proposed system, utilizing an ensemble machine model and three Pentacam corneal indices, aims to assess KC severity before visual impairment occurs in a timely manner. A user-centered, iterative development methodology [44] is employed to build the proposed system, ensuring the ongoing engagement of potential end-users (ophthalmologists) throughout the development process. A transparent approach based on expert opinion is adopted to feature selection, model development and validation tests. This facilitates regular updates to models based on new data and continuous monitoring of the system’s performance. The primary contributions of this study include: (i) a comprehensive approach to collecting and pre-processing a raw clinical dataset, (ii) the proposal of a severity staging system (0–4) based on only three corneal tomography parameters, (iii) the development and evaluation of multiple classification models capable of detecting various levels of KC severity, and (iv) the creation and deployment of a real-world online decision support system. This system aims to standardise the diagnostic criteria for KC severity across multiple eye-care facilities, thereby reducing the potential for human error, especially in geographical regions lacking specialist ophthalmologists. This research extends the outcome of an earlier study [45], carried out by the authors, which focused on the classification between normal and KC corneas. In this work, the emphasis is specifically on classifying various severity stages of KC.

## Methods

### System overview

**Fig. 1:**
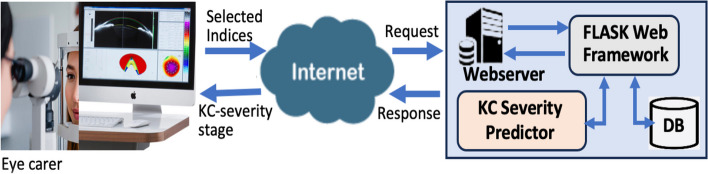
Workflow of the user-system interaction

The primary objective of the proposed system is to aid general practitioners, particularly those located in underserved geographical areas, in the screening for KC severity. Figure 1 depicts a streamlined workflow diagram illustrating the interaction between the user and the system, briefly outlined as follows: The user manually collects several corneal indices from a Pentacam imaging device and submits them to the system through a browser on a computing device, such as a laptop, tablet, or smartphone. The Flask web framework receives and processes the user's request. In response to this request, the Flask framework manages the input and produces a predicted KC severity level based on the received set of corneal indices.

The detection of the severity stage is performed by a ML model aided by an SQLite database that functions as a repository for user inputs, associated predictions, and user access credentials. This information can later be utilized for tracking disease progression and as additional training data to enhance the prediction accuracy. Subsequently, the web server communicates the prediction result to the user by delivering it to the user's browser, which then presents the result on the screen of the computing device in use.

### Development methodology

**Fig. 2:**
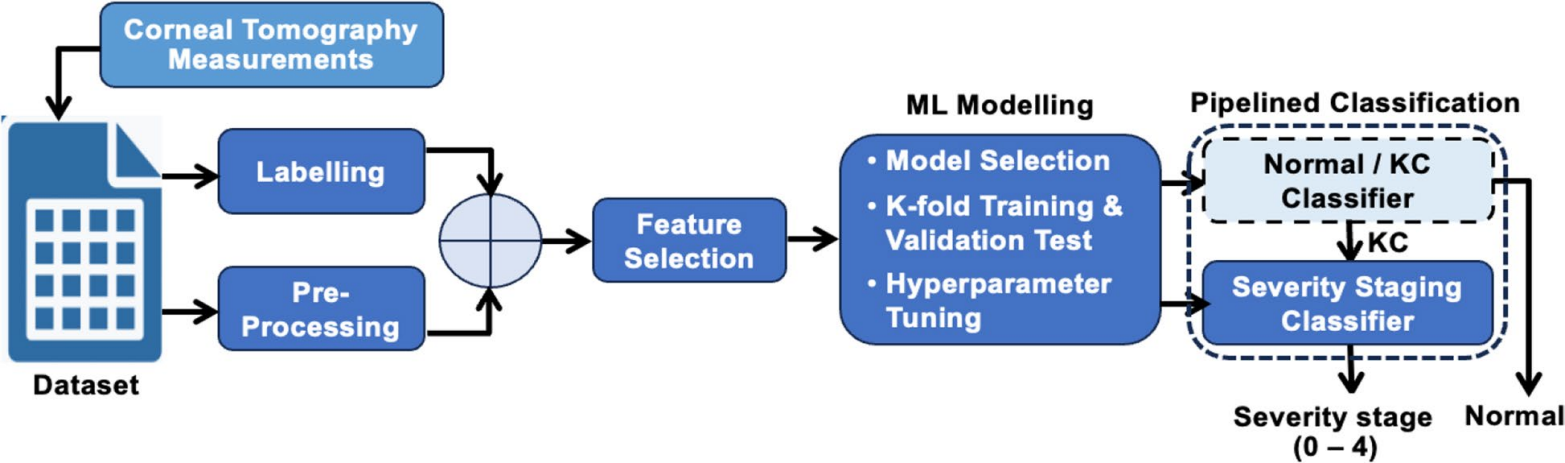
Development stages of the proposed staging predictor

The key phases in the development methodology of the proposed severity staging predictor are shown in Fig. 2. The process starts with the extraction of the study dataset from Pentacam [46]. Pentacam is a corneal imaging device incorporating a slit illumination system and a camera that rotates jointly around the eye. The slit illuminates a thin layer within the eye, and due to their lack of complete transparency, the cells scatter the slit’s light. Next, the collected data is pre-processed and labelled by a team of ophthalmologists.

A subset of several indices (features) is then identified to differentiate between the different severity levels of the disease. The identified features are then employed to create ML models that are pipelined (Fig. 2). It is worth noting that the classifying model of normal/KC corneas, as previously detailed by the authors [45], is beyond the scope of this paper. This study specifically focuses on the severity staging classifier (KC severity predictor). To enhance accessibility and standardize the diagnosis criteria across multiple eye-care facilities, a web interface was built and utilized to deploy the developed severity predictor on a web application server. The development methodology for the proposed system is presented and discussed later in the ML modelling section.

### Study dataset

**Fig. 3:**
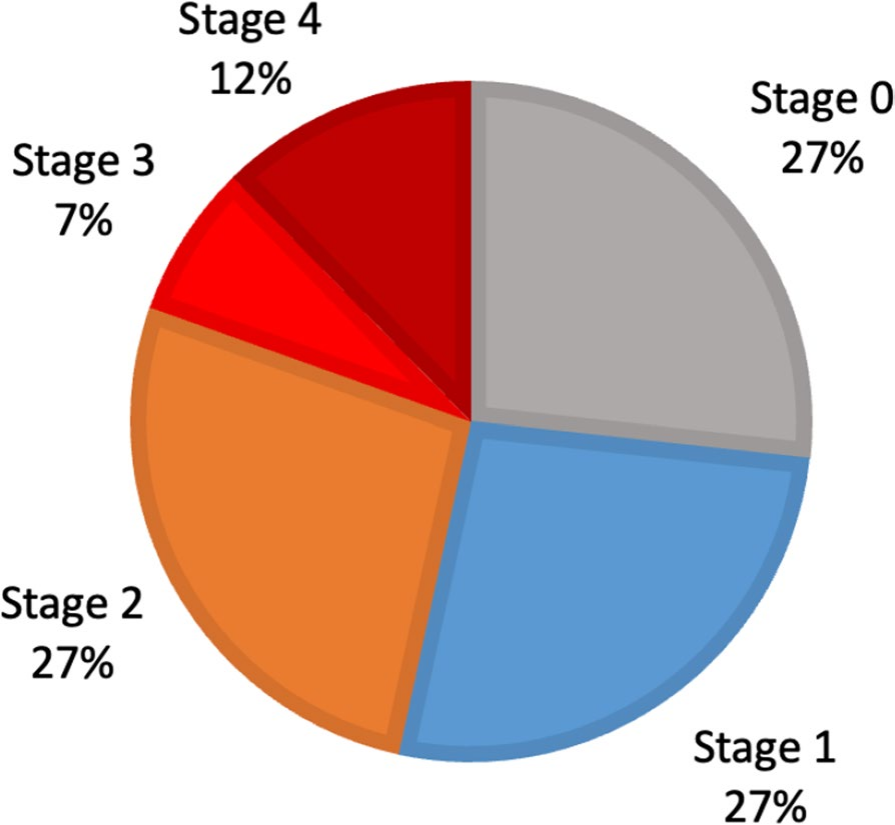
Sampling distribution of the collected dataset (*n* = 644)

The dataset utilized in this study was collected over the preceding decade from two eye-care centers in Jordan: Jordan University Hospital (JUH) and Al-Taif Eye Center (ATEC). Ethical approval for the study was obtained from the Ethics Committees at both healthcare facilities (Protocols: JUH-2023–1593/67 and ATEC-GM/15). The dataset consisted of patients with a diagnosis of KC in one or both eyes. Diagnosis was established through clinical, optometric, and ophthalmic examinations, including slit-lamp assessment, retinoscopy, and corneal tomography data. The collected dataset, comprising 79 feature columns linked to 644 corneas with different severity stages, is shown in Fig. 3.

As illustrated, the dataset samples exhibit an imbalanced sample distribution among the various stages of KC severity. This imbalance, which is common in medical research [47, 48], can lead to biased classification. Consequently, it is imperative to address this concern prior to training ML models to prevent potential biases in both training and classification performance.

### Pre-processing

**Fig. 4:**
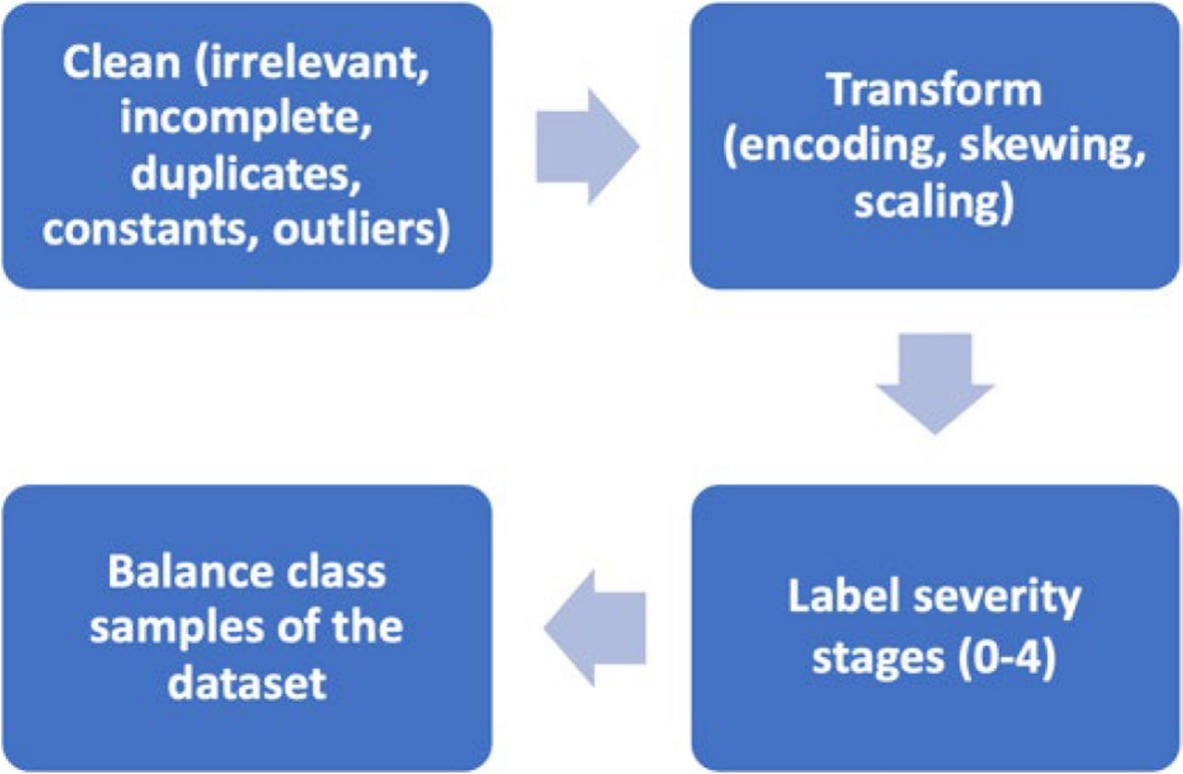
Pre-processing procedures applied to the study dataset

In this study, several pre-processing procedures were applied to the raw data to enhance its quality thereby improving the performance of the feature selection and ML modelling processes. These procedures are shown in Fig. 4 and are detailed as follows.

#### Data cleaning

Table 1 outlines the steps that are applied to the raw dataset, resulting in a reduction of feature columns from 79 to 58. Handling poor-quality data is essential in ML modelling; the Expectation–Maximization (EM) algorithm [49, 50] is one of the widely used iterative methods for finding maximum likelihood or maximum posteriori estimates of parameters in statistical models. However, in the collected study dataset, the feature columns containing incomplete data are found to be irrelevant to the intended diagnosis, and thus were identified and safely filtered with the aid of expert ophthalmologists.

**Table 1:** Outline of the implemented data cleaning procedures

Procedure	Description
Irrelevant data	The dataset is purged of any data points considered irrelevant for the intended diagnosis
Poor-quality data	Feature columns containing incomplete data are filtered out because most ML models cannot handle missing values
Data replication	Redundant elements, which share the same value, are dependent on other parameters, or are derived from them, are eliminated from the dataset in close consultation with ophthalmologists
Constant values	Feature columns with constant values are excluded as they lack informative content that could assist the ML model in distinguishing between various disease conditions
Outliers	Data values that significantly deviate from other data elements are filtered out

Identifying outliers often requires statistical methods or domain expertise [50]. Common approaches include standard deviation, median absolute deviation, z-score, boxplot and ML techniques like clustering and anomaly detection algorithms. The boxplot [51], which relies on the interquartile ange (IQR), is adopted in this study due to its interpretability and effectiveness in identifying outliers within small datasets [52]. Its strength lies in its resilience against extreme values, offering a more reliable measure than methods relying solely on mean or standard deviation. This is particularly beneficial for small datasets where outliers can disproportionately influence these traditional measures. Outliers are identified as observations falling below a lower bound = Q1 − k × IQR or above an upper bound = Q3 + k × IQR, where k = 1.5, and Q1 and Q3 represent the first and third quartiles, respectively [53].

#### Feature transformations

Several feature transformation techniques are implemented on the study dataset, encompassing the encoding of categorical data, skew transformation, and feature scaling. These techniques are briefly described as follows.

##### Feature encoding

Involves the conversion of non-numeric values to numeric values, a process commonly applied to categorical features representing qualitative data without inherent mathematical meaning. While easily comprehensible to humans, such data poses challenges for computers. Consequently, all categorical data are transformed into numerical data types. Binary or one-hot encoding (0, 1) is employed for nominal (categorical, unordered) features, while ordinal encoding (1, 2, … n) is utilized for ordered (categorical, ordered) features. For instance, numerical values (0–4) are used to replace diagnosis labels indicating severity stages (0–4).

##### Skew transformation

raw datasets often exhibit positive skewness (peaking to the right) or negative skewness (peaking to the left), deviating from a normal distribution. Numerous statistical tests, including ANOVA, F-test, and others, require data to have a normal or near-normal distribution. The current dataset exemplifies such asymmetry, with skew values ranging from 3.33 to − 15.47; values notably outside the acceptable range of typical statistical tests (+ 2 to − 2) [54]. It becomes imperative to eliminate this skewness, bringing the dataset as close as possible to a normal Gaussian distribution. After experimenting with multiple transformations including the log, Box-Cox, square root (SQRT) and others, the SQRT was identified as the most suitable method to bring all skewed features within the acceptable range.

##### Feature scaling

Prior to training the proposed models, data normalization is employed on the dataset to mitigate distortions arising from features with disparate scales, facilitating improved interpretation of distance-based approaches. Various methods exist to normalize feature values, ensuring they are measured on a consistent scale. Common techniques include min–max scaling, mean scaling, and standard scaling. In this study, the latter two methods, which can normalize both positive and negative feature values to be within the range of − 1 and + 1—consistent with the characteristics of the study dataset—are explored. Results indicated that both techniques exhibit comparable performance in most cases, with the standard method slightly outperforming in the remaining instances, and thus the standard scaling method was adopted.

#### Labelling severity stages

A team of specialist ophthalmologists labelled the collected subjects using clinical examinations, slit-lamp assessments, and corneal topography data from Pentacam imaging devices. Pentacam exhibits the highest repeatability, establishing its effectiveness as a tool for KC severity classification and monitoring KC progression [42]. After applying the labelling criteria, the study subjects were categorized into five severity stages (0–4). Concise definitions for these stages are outlined in Table 2, accompanied by a representative image of the Sagittal curvature (front) corresponding to each level.

**Table 2:** Concise definitions of keratoconus severity stages [55, 56]

Representative Image	Description
	Stage 0 – clear cornea with normal thickness and corrected distance visual acuity (CDVA) of 6/6
	Stage 1 – clear cornea with the potential presence of Fleischer's ring, mild corneal thinning evident on topography but not grossly, distorted reflex on retinoscopy, and CDVA less than 6/6
	Stage 2 – Fleischer’s ring and Vogt’s striae, corneal thinning may be evident grossly, scissoring reflex on retinoscopy and CDVA below 6/12
	Stage 3 – Initial manifestation of Munson's sign, significant corneal thinning with faint scarring, retinoscopy difficult to perform, spectacles distance visual acuity worse than 6/30, yet there is potential improvement to 6/6 with contact lenses
	Stage 4 – corneal scarring and opacities at the apex, evident Munson's sign, retinoscopy impossible to perform, CDVA worse than 6/120 and not achieving 6/6 even with contact lenses

#### Balancing class sampling

Addressing the uneven distribution within a dataset can be approached through various methods, such as oversampling minority classes, undersampling majority classes, or employing a combination of both strategies. In this study, the latter approach was adopted as follows. For the severity staging, where the available number of samples was relatively limited, the minority class samples for Stage 3 and Stage 4 were oversampled to achieve a reasonable balance with the samples from the remaining classes of Stage 0 to Stage 2. This is accomplished through the application of Synthetic Minority Oversampling TEchnique (SMOTE). SMOTE, known for its simplicity and effectiveness in addressing imbalances in small-sized datasets [57–59]. It generates data points along the line segment between a randomly selected data point and one of its K-nearest neighbours.

**Fig. 5:**
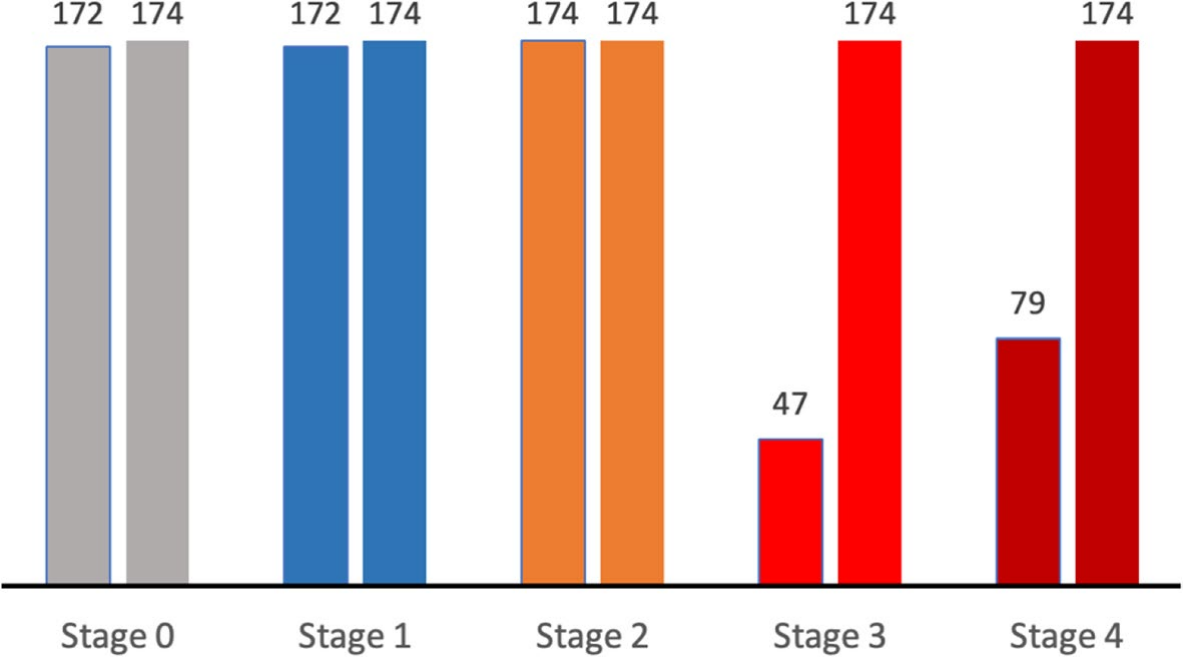
Comparison between real samples (left columns) and augmented samples (right columns) in each stage

Following the implementation of SMOTE, the minority classes of stages 0, 1, 3, and 4 were augmented to match the majority class samples (174) of Stage 2. As a result, the dataset was boosted from 644 to 870 samples, with 174 samples per class. Figure 5 presents a comparison between the real samples (left columns) and augmented ones (right columns) in each stage. These adjustments were anticipated to enhance the training and classification performance of the proposed models and mitigate the adverse effects of a small sample size.

### Feature selection

The proposed feature selection process involved analysis of feature-relative importance and feature dependency using a combination of expert opinion, probability, and visual methods.

#### Feature dependency

Certain features, which either directly or indirectly rely on primary features have been identified with the aid of ophthalmologists. These features include [60]:

- RSagMin depends on R_Min (mm).
- R_Min (mm) and R_Min_B (mm) depends on KMax_Sag_Front (D).
- Rs _B (mm) and K2_B (D) are dependent on one another.
- K2_F (D) and Rs_F (mm) are products of one another.
- Km_B (D), K1_B (D) are dependent on Rf_B (mm).
- K1_F (D) and Km_F (D) are dependent on Rf_F (mm).

After filtering these features and others, the feature set was reduced from 58 to 40 features.

#### Feature relative importance

In ML, feature importance entails assigning scores to input features in a predictive model, indicating their relative significance in the prediction process. These scores are relevant to both regression problems, focused on predicting numerical values, and classification problems, where the objective is to predict class labels, as is the case in this study. It should be mentioned here that the feature importance is a relative measure within the context of the model and the specific dataset used for training.

**Fig. 6:**
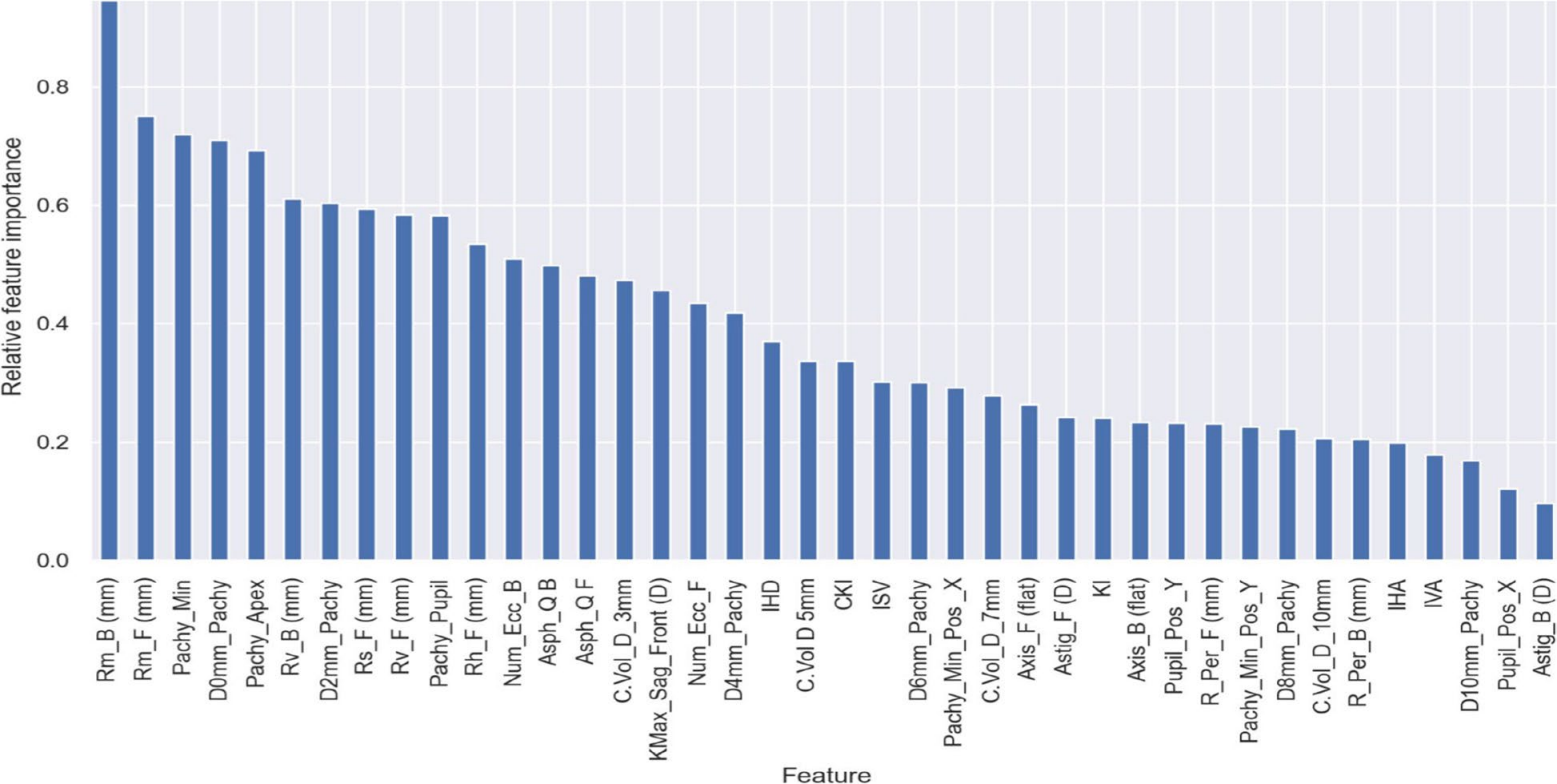
The relative importance of features within the dataset for predicting the severity class labels based on the Gini method (*n* = 40). Asph_QB, asphericity coefficient (Q value) of the corneal back surface (posterior), asphericity Q value refers to the variation in the curvature of the cornea from its center to the periphery; Asph_QF, asphericity coefficient (Q value) of the corneal front surface (anterior); Astig_B (D), central corneal astigmatism (posterior corneal values measured in diopters); Astig_F (D), central corneal astigmatism (anterior corneal values measured in diopters); Axis_B (flat), corneal meridian of the least astigmatic power (posterior); Axis_F (flat), corneal meridian of the least astigmatic power (anterior); CKI, central keratoconus index; D0mm_Patchy – D10mm_Pachy, average pachymetry on concentric rings with radii (0–10 mm) around corneal thinnest point, respectively; IHA, index of height asymmetry; IHD, index of height decentration; ISV, index of surface variance; IVA, index of vertical asymmetry; KI, keratoconus index; KMax_Seg_Front (D), keratometry of the steepest point (anterior); Num_Ecc_B and Num_Ecc_F, Fourier-based posterior and anterior eccentricity in central 30 degrees, respectively; Pachy_Apex, corneal thickness in apex; Patchy_Min, thinnest pachymetry (µm); Pachy_Min_Pos_X and Pachy_Min_Pos_Y, x- and y-coordinates of the thinnest location, respectively; Pupil_Pos_X and Pupil_Pos_Y, x- and y-coordinates of the pupil position relative to the corneal apex, respectively; Pachy_Pupil, corneal thickness at the pupil center; Rh_F (mm), central radius in horizontal direction (anterior); Rm_B (mm), curvature radius of the back surface of the cornea (posterior); Rm_F (mm), curvature radius of the front surface of the cornea (anterior); Rs_F (mm), steepest radius (anterior); R_Per_F (mm), average anterior radius of curvature between 6 mm and 9 mm zone; R_Per_B (mm), average posterior radius of curvature between the 6 mm and 9 mm zone; Rv_B (mm), central radius in vertical direction (posterior); Rv_F (mm), central radius in vertical direction (anterior)

In practical applications, various ML libraries, including the scikit-learn library in Python, offer a “*feature importance”* attribute once a random forest (RF) classification model has been trained. In this model, a common method (called Gini), was utilised for calculating the feature importance scores. It is based on the Gini impurity reduction achieved by each feature. Although Gini impurity is not a conventional statistical test, it is a concept rooted in probability and information theory. This concept finds extensive application in ML, particularly in the construction of decision trees and the evaluation of feature importance within RF. The Gini method was applied to the remaining 40 features, resulting in their prioritization based on importance scores (Fig. 6).

The features with the top three scores are selected and employed in this study to create different ML models aimed at detecting distinct stages of KC severity. These features are: (i) the corneal posterior radius of curvature, Rm_B (mm), (ii) anterior radius of curvature, Rm_F (mm), and (iii) the thinnest pachymetry, Pachy_Min, attained relative importance scores of 0.938, 0.745, and 0.734, respectively. These scores serve as a valuable tool for identifying and prioritizing features based on their significance in the classification task (i.e., KC severity staging). Other features with slightly lower scores were often dependent on or derived from these core indices. For instance, the average pachymetry on concentric rings with radii 0 mm (D0mm_pachy) around the thinnest point of the cornea is technically the same as Pachy_min, and thus it was excluded to maintain clarity and prevent redundancy. It should be noted here that all the selected features were derived from a single corneal imaging device (Pentacam).

#### Visualisation

**Fig. 7:**
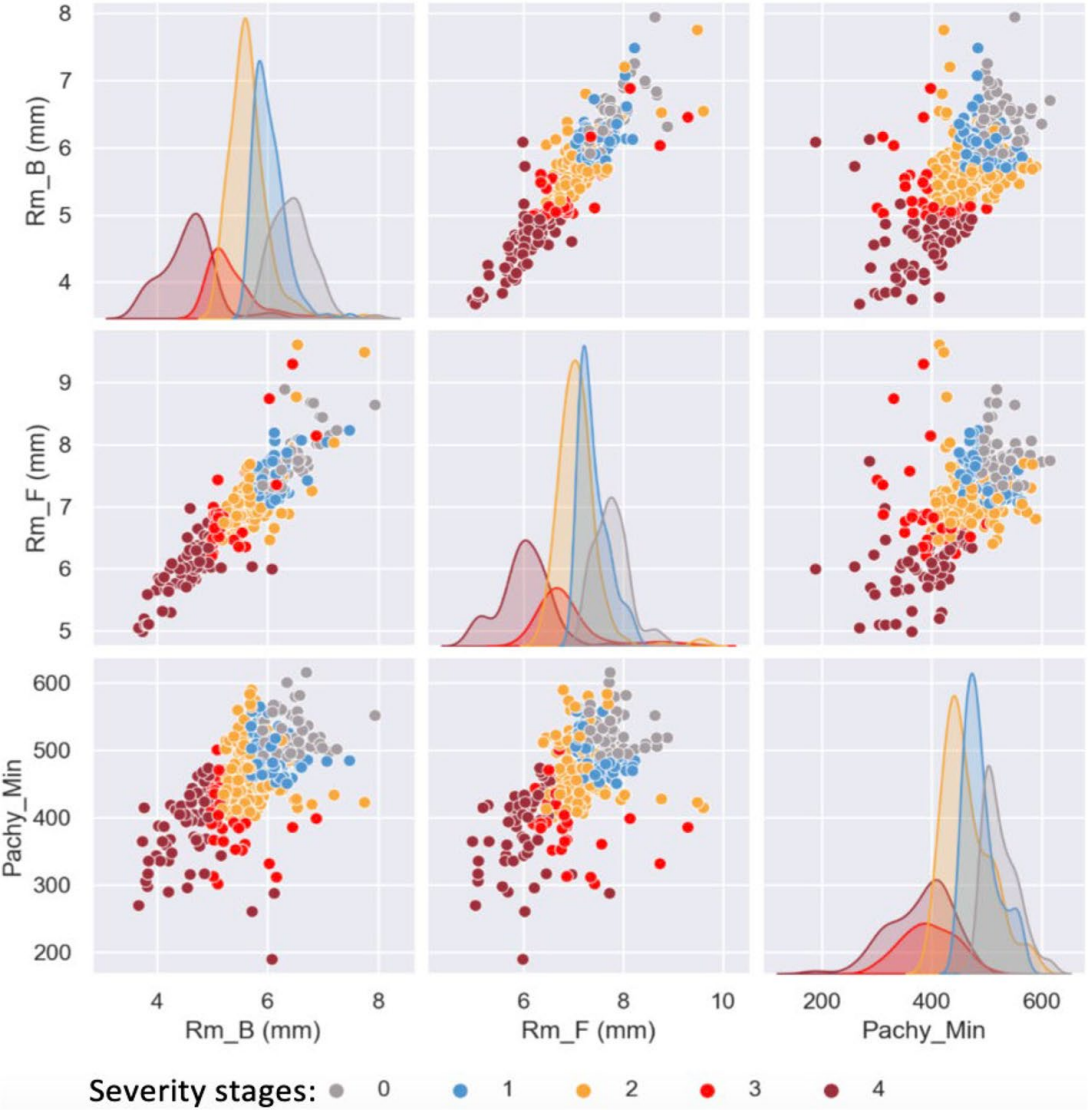
Pairwise bivariate distributions of the selected features. Rm_B (mm), curvature of the back surface of the cornea (posterior), measured in mm; Rm_F (mm), curvature of the front surface of the cornea (anterior), measured in mm; Patchy_Min, thinnest pachymetry measured in µm

To better understand the relationships between the identified top features, a Python library called Seaborn, was utilised to generate multiple pairwise bivariate distributions using a pair plot (Fig. 7). This plot enables the visualization of individual feature distributions and the relationships between two features in the dataset. The univariate histograms for every feature were generated in the diagonal plots to illustrate the marginal distribution of the data in each column. Examining the diagonal as well as non-diagonal relationships between features helped to identify which feature pair will have the best separation between the target classes (i.e., severity stages). As illustrated, the *Rm_B (mm)* is more effective in separating the different severity classes than the *Rm_F (mm)* and *Pachy_Min*. This validates the significance of the selected features.

### Machine learning modelling

**Fig. 8:**
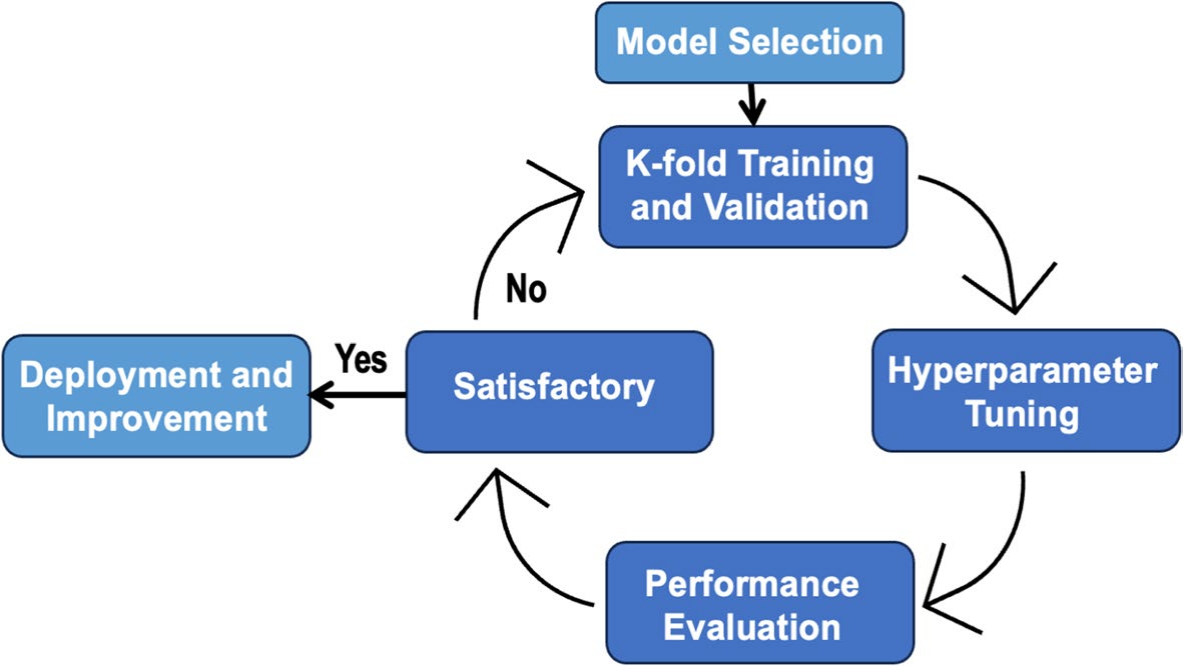
The development process method

A user-centered, iterative approach [44] was applied in the development of the proposed system, ensuring the continuous involvement of potential end users throughout the process. Figure 8 illustrates a simplified flow diagram of this process, with its distinct phases briefly described as follows.

#### Model selection

To establish the end-to-end configuration and validate the concept of the proposed ML solution, simple models can be utilized. This helps prevent excessively complex designs, reduces the time it takes to implement a solution [43], and may mitigate the potential risk of overfitting. Following the pre-processing of the dataset and identifying the most relevant subset of features for the target variable (i.e., severity stages), a classification model was chosen. This selection was made through experimentation and performance comparisons of three popular ML models in KC detection including severity staging. These models were logistic regression (LoR), support vector machines (SVM), and ensemble RF. These models are implemented using the Anaconda Jupyter notebook [61]. The fundamental principles underlying these classification models are briefly described as follows.

##### Logistic regression (LoR) classifier

It is a probabilistic classification model that employs the Sigmoid function and limits the probability values to a range between 0 and 1. If the predicted value exceeds a specified threshold, the event is considered more likely to occur, while if it falls below the threshold, it is deemed less likely to occur [62]. However, to apply LoR to multi-class classification, we utilized an extension known as multinomial LoR. This extension provided native support for addressing the five-class severity staging under investigation.

##### Support vector machine (SVM) classifier

It divides the various classes within the training set into groups using a surface that maximizes the margin between each class. The objective of SVM classification is to create lines that effectively partition the data points. The aim is to identify the optimal line i.e., one that maximizes the margin between the classes [63, 64]. SVM is well suited for binary classification problems but for multi-class challenges, a technique known as "one-versus-one" (OVO) is employed, wherein each class is matched against every other class. In the final stages of classification, during the testing phase, a single vote is cast for the predominant class in each classification. The class assigned to the test dataset is then determined by the highest number of votes.

##### Ensemble random forest (RF) classifier

It employs an ensemble approach, combining individual decision tree learners into a "forest" to enhance overall strength while maintaining a balance between robustness and prediction accuracy [45]. The process involves generating numerous trees, and for each tree within the training set, the bootstrap aggregation (bagging) method is employed. Every tree in the forest receives input from the categorization algorithm, contributing a separate vote for each class. The ultimate class determined by the RF is the one with the highest vote count [65]. Furthermore, the RF maintains some distinction at each node when splitting similar features [66, 67].

### K-fold training and validation

**Fig. 9:**
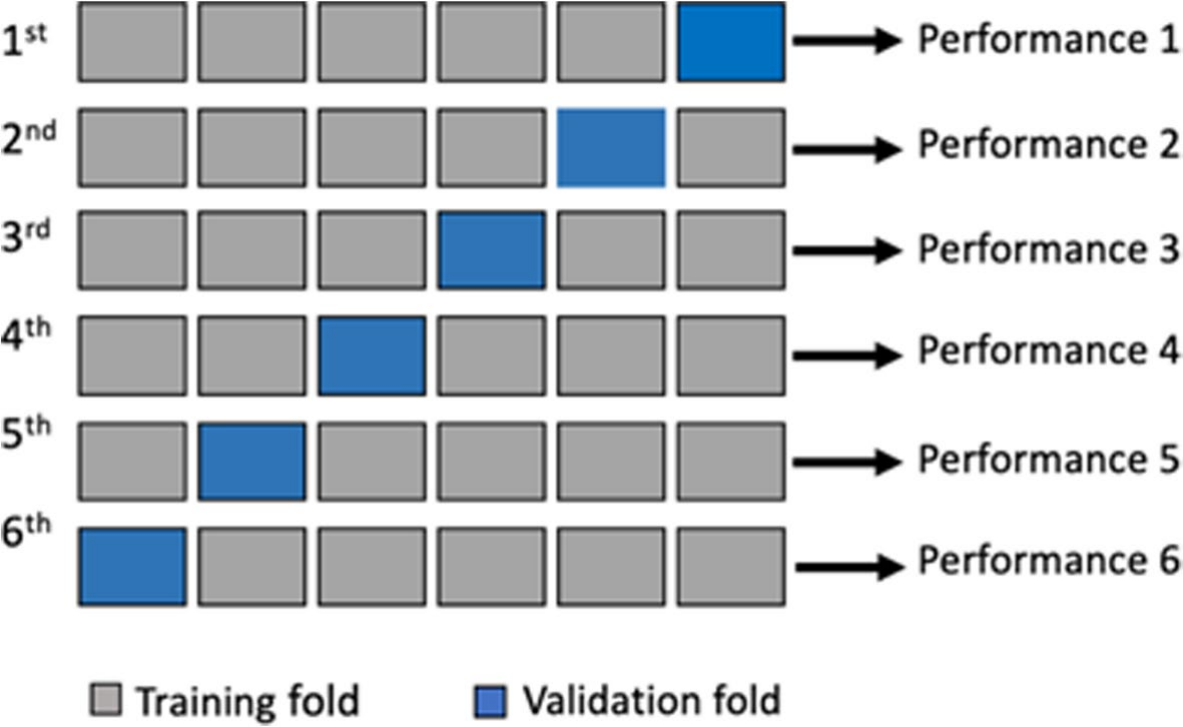
Six-fold cross validation

This study utilizes k-fold cross-validation to reduce the influence of the specific selection of test and training data on model evaluation. It involves creating non-repetitive subsets from the training data. The study dataset was divided into six folds based on the optimal performance observed across various k-fold divisions. Specifically, five folds (83.33%) were utilized for training, and the remaining fold (16.67%) was reserved for validation. This iterative process was repeated six times, with a distinct fold designated for validation in each iteration, as illustrated in Fig. 9. The trained classifier was subsequently tested and validated using evaluation metrics, and the results were averaged over four runs. The average performance is calculated using Eq. 1, as follows:

1 $$Performance \left(ave\right)=\frac{1}{6} \sum_{i=1}^{6}Performance \left(i\right)$$

### Hyperparameter tuning

**Fig. 10:**
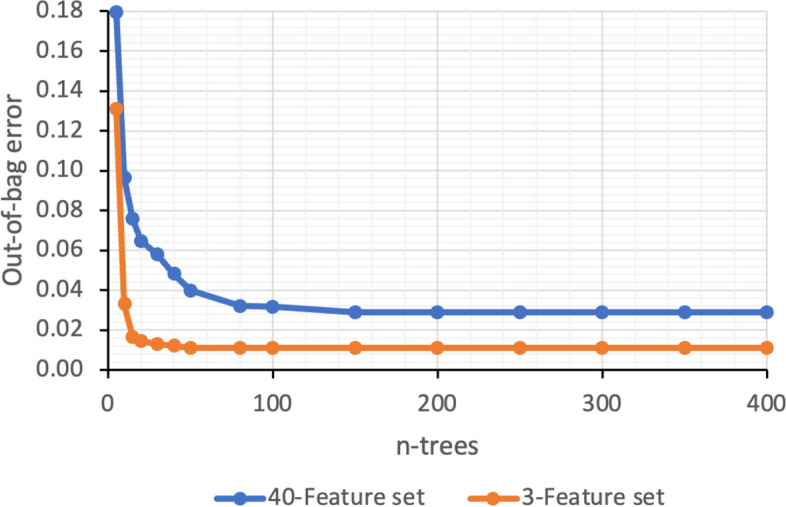
Out-of-bag error versus number of trees

In RF, the number of estimators (n-estimators) serves as a crucial hyperparameter for bagging trees. Thus, minimizing the out-of-bag error involves tuning this parameter. The process begun with the use of two trees, and more were gradually added until the out-of-bag error stabilized at a specific minimum number of trees. In this experiment, both the model with the selected 3-feature subset and the 40-feature set were employed to determine the optimal number of trees. As depicted in Fig. 10, the optimum number of trees was 150 for the 40-feature set and 50 for the 3-feature subset, beyond which the out-of-bag error curve flattens. Notably, utilizing the selected feature subset had resulted in a reduction of 66.66% in the number of trees. Similarly, the model's training time was also reduced by less than 30% compared to the time required for the 40-feature set.

The tuning of both the number of trees and other parameters of the RF model was also experimented through two distinct methods: GridSearchCV (GSCV) and RandomSearchCV (RSCV). GSCV extensively explores a prespecified set within the targeted model's hyperparameter range [68, 69] while RSCV uses a probability distribution to assign a value to each hyperparameter individually [70], making it notably faster than GSCV. However, the results obtained from the GSCV method exhibited greater consistency with the number of estimators obtained from Fig. 10, resulting in enhanced performance. Tuning parameters of both the LoR and SVM classifiers were experimented using both GSCV and RSCV methods. Likewise, the parameters tuned by GSCV for both classifiers resulted in better performance compared to those obtained by the RSCV method. As a result, GSCV was employed to fine-tune the parameters of all the implemented models. The main parameters of the implemented models are given in the Appendix (Tables A. 1, A. 2 and A. 3).

**Table 3:** Performance comparison of the developed models

Performance metric (%)	LoR Model	SVM Model	RF Model
Accuracy	73.80	80.00	98.62
Precision	73.21	79.49	98.70
Sensitivity	73.80	79.67	98.62
F1-score	73.5	79.58	98.66
F2-score	73.68	79.63	98.64

*LoR = *logistic regression; *SVM = *support vector machines; *RF = *random forest

## Results

A confusion matrix is a commonly used graphic for evaluating the performance of a specific classification and is employed to assess the effectiveness and robustness of the developed models. The ground truth (target classes) is represented on the x-axis of the matrix, while predicted classes are represented on the y-axis. True positive (TP) corresponds to situations where both the predicted and actual class values are 1. True negative (TN) indicates that both the expected and actual classes have a value of 0. When the anticipated class differs from the actual class, false negatives (FN) and false positives (FP) occur.

**Fig. 11:**
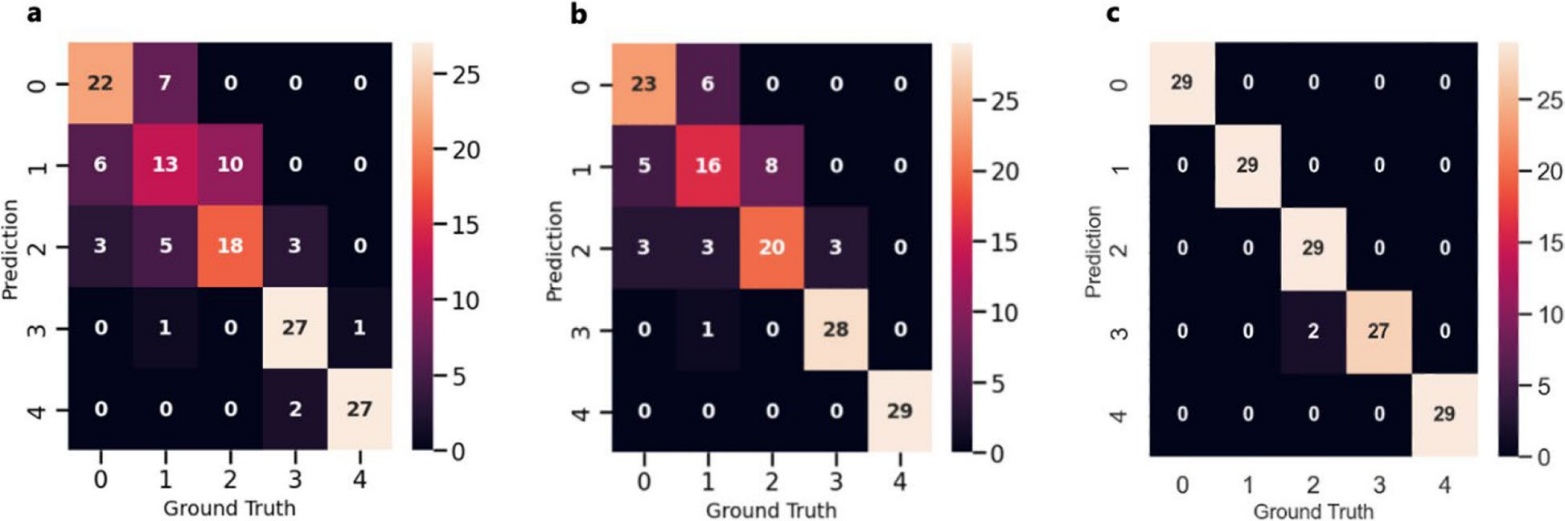
Confusion matrixes of the developed classifier models. **a** Logistic regression; **b** Support vector machine; **c** Random forest

The results presented in the confusion matrices of Fig. 11 that are utilized to assess performance of the created models, are computed using Eqs. 2,3, 4, 5 and 6 as follows:

*Accuracy* – the ratio of accurate predictions to the total number of input samples, calculated as:

2 $$Accuracy=\frac{TP+TN}{TP+TN+FP+FN}$$

*Precision* – the average percentage of the actual positive cases among the retrieved instances, calculated as:

3 $$Precision=\frac{TP}{TP+FP}$$

*Sensitivity (or Recall)* – the percentage of actual positive cases that were correctly predicted, calculated as:

4 $$Sensitivity (or Recall)=\frac{TP}{TP+FN}$$

*F1-score* – the sensitivity and precision of the system are both considered in the calculation of this score:

5 $$F1-score =2\times \left(\frac{Precision\times Sensitivity}{Precision+Sensitivity} \right)$$

*F2-score* – the precision- and sensitivity-weighted harmonic mean (given a threshold value), calculated as:

6 $$F2-score = 5\times \left(\frac{Precision\times Sensitivity}{\left(4\times Precision\right)+Sensitivity}\right)$$

In contrast to the F1-score, which assigns equal importance to precision and sensitivity, the F2-score diminishes the significance of precision while amplifying the importance of sensitivity. As a result, it places greater emphasis on minimizing FN rather than minimizing FP. Table 3 presents the average performance outcomes for predicting the severity stages in the developed models. As evident, the RF model exhibited superior performance compared to both the SVM and the LoR. Therefore, in the context of distinguishing between different levels of KC severity, the ensemble RF model was employed as a predictor within the proposed system.

### Model deployment and improvement

**Fig. 12:**
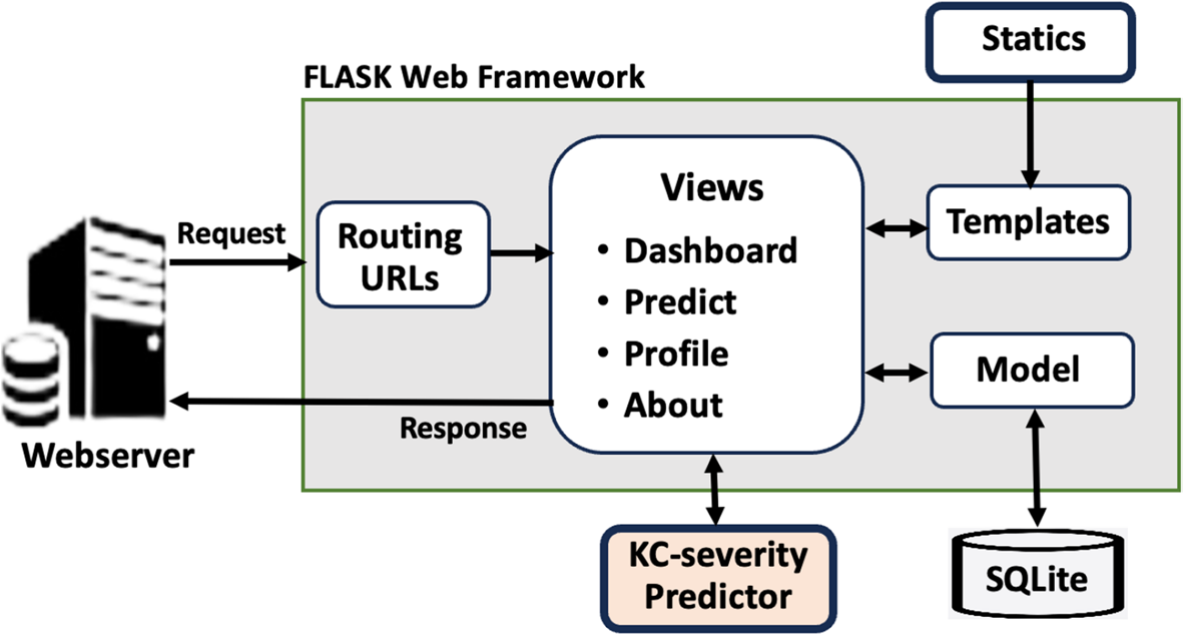
Structure overview of the flask framework

To assess the developed model in a real-world setting, it needs to be incorporated into the necessary software infrastructure for execution. This process encompasses integration, monitoring, and updates post-initial deployment. The integration of the model comprises two essential tasks: setting up the infrastructure for model execution and implementing the model itself. To achieve this, a lightweight Flask web framework [71] was employed to construct the interface essential for incorporating the developed KC predictor. Flask facilitates the development of online applications using Python, equipped with various libraries and frameworks, especially suitable for projects involving artificial intelligence. The primary resources of Flask utilized to craft the web interface for the proposed system are depicted in Fig. 12 and briefly outlined in Table 4.

**Table 4:** Key components of the flask web framework [72, 73]

Component	Description
Views	A class that allows for the creation of several instances with varying arguments. It can be utilized to modify the behaviour of the view. In the current prototype, this class is connected to the *app.route* decorator that loads the required data onto a web page and displays it
Routing URLs	It associates URLs with operations like serving pages or data. The created prototype makes use of static route URLs. However, more sophisticated applications can also make use of dynamic route URLs
Statics	A subdirectory contains the application's JavaScript and cascading style sheets (CSS). As a result, users can access these files using the secure HTTP extension (HTTPS) and the Hypertext Transfer Protocol
Templates	Provides various types of data files, including photos, Java Scripts, or cascading style sheets (CSS). It offers static file management [74, 75]. The current prototype also makes use of Bootstrap to adjust the webpages to fit different screen sizes
Model	Flask can be used with and without database. In this prototype, the SQLite database is employed to temporarily store the user inputs and the relevant predictions together with the user's access credentials

The ML community is still facing challenges in monitoring and updating ML systems [76]. For example, they are still learning what data and model metrics are most important to track and how to set off alarms on the system when abnormal behaviour is detected [42]. The optimal methods for monitoring changing input data, addressing prediction bias, and evaluating the overall performance of ML models remain unclear. Furthermore, ensuring that the model consistently reflects the latest developments in data and the environment often necessitates the ability to update the model post-initial deployment. Several methods exist for updating models with new data, including continuous learning and regularly scheduled retraining. A crucial factor influencing the frequency and quality of the model update process is concept drift, commonly referred to as dataset shift [77].

## Discussion

### Clinical classification

Several classification schemes for KC severity have been reported in the literature [78–86]. The AK classification system, one of the earliest systems, categorizes the severity of KC into four stages. It considers factors such as spectacle refraction, central keratometry, the presence or absence of scars, and central corneal thickness [87]. To improve the classification of disease severity, others have made modifications and additions to this classification [56, 88]. Alongside these classification systems, having a standardized method for documenting the progression of ectasia is crucial. The decision to recommend treatments such as corneal cross-linking heavily depends on well-documented ectasia progression in clinical assessments.

The 2015 global consensus that was published by a committee of expert ophthalmologists [21, 89] concluded that "abnormal posterior ectasia, abnormal corneal thickness distribution, and clinical non- inflammatory corneal thinning are mandatory findings to diagnose keratoconus." However, this definition is not easy to implement because the agreement did not specify thresholds or parameters for diagnosing KC including its severity stages, and thus it is still subject to different interpretations. In the studies of Duncan et al. [90, 91], the authors proposed an ABCD classification system that scores KC severity from 0 to 4. More recently, in response to limitations in the AK system and guided by the global consensus document on KC and ectatic diseases, Belin et al. [92, 93] introduced a new ABCD severity staging system. The utilization of this system on Pentacam (Oculus GmbH, Wetzlar, Germany) [46] was motivated by its high measurement repeatability, surpassing that of other corneal imaging devices [94].

Each of the reported classification systems provides unique insights into the extent, location, and clinical signs of KC, contributing to a comprehensive evaluation of disease severity. In this study, the subjects in the study dataset were therefore graded utilizing a combination of clinical examinations, slit-lamp assessments, and corneal topography data obtained from Pentacam imaging devices, as detailed in the section on pre-processing. The classification results from the ML predictions demonstrated a strong correlation with the clinical classifications. This confirms the validity and effectiveness of the developed ML model.

### Feature selection

Experimentation involved a raw clinical dataset comprising 644 subjects (augmented to 900 samples, with 180 samples per class), and 79 feature columns. After several data cleaning steps, the feature columns were reduced to 58 features. Subsequently, a feature selection process involved feature-relative importance and feature dependency analysis was implemented. A combination of expert opinion, probability, and visual methods were employed to narrow down the features to a subset of only three, representing a mere 3.79% of the total raw dataset features.

The significance of this selected feature subset, characterized by high relative importance, was validated through both visual observations (depicted in Fig. 7) and the consensus of domain experts. This confirmed the reliability and effectiveness of the implemented pre-processing and feature selection process. The significance of the selected features in the classification of KC severity are outlined as follows:

*Posterior radius of curvature (PRC) in the 3.0 mm zone*, represented by Pentacam’s *Rm_B (mm)* parameter. It measures the curvature of the posterior (back) surface of the cornea. This measurement is critical for assessing the shape and structure of the cornea, playing a pivotal role in the assessment of KC severity, which involves structural changes in the posterior corneal surface. In the relative importance analysis presented in Fig. 6, the PRC attained the highest ranking, scoring 0.938.

*Anterior radius of curvature (ARC) in the 3.0 mm zone*, denoted by Pentacam’s *Rm_F (mm)* parameter. It measures the curvature of the cornea's anterior (front) surface. This measurement holds significance in evaluating the shape of the cornea and is frequently considered in the assessment of overall corneal condition including KC severity. ARC secured the second-highest position in the relative importance analysis, achieving a score of 0.745, as shown in Fig. 6.

*Thinnest pachymetry measured in µm*, represented by Pentacam's *Pachy_Min* parameter. It offers insights into the minimum thickness at a specific point called the thinnest location. This measurement is crucial for assessing the severity of KC, where variations in corneal thickness are indicative of the condition's progression and severity. In the feature selection analysis, this parameter ranked third with a score of 0.734 (Fig. 6).

Table 5 presents median values of the selected features, and these values correspond to the thresholds specified in Belin's ABCD grading system for the respective severity levels [92, 93]. However, Belin's system also considers the best-corrected visual acuity (BCVA) in addition to the features identified in this study. The BCVA is obtained through an optometric refractive examination and remains independent of corneal topography. Also, it should be noted that this set of features is distinct from the subset that was previously identified in [45] for the classification of normal and KC corneas.

**Table 5:** Median values of the selected features for different severity stages

Severity	PRC (mm)	ARC (mm)	Thinnest pachymetry (µm)
Stage 0	6.4	7.7	518.0
Stage 1	6.0	7.3	480.5
Stage 2	5.6	7.0	448.0
Stage 3	5.1	6.7	391.0
Stage 4	4.6	6.0	395.0

*PRC = *posterior radius of curvature; *ARC = *anterior radius of curvature

### Model classification performance

The clinical dataset employed in this research was gathered and validated by ophthalmologists and underwent meticulous pre-processing to ensure consistency throughout the training and validation phases. Table 6 presents a comparison between the proposed system and state-of-the-art methods, considering various common performance indicators. This comparison also encompasses information related to the models used, dataset sizes, input data types, as well as the number of input features (parameters) used.

**Table 6:** Comparison with state-of-the-art KC severity staging techniques (as of 2018)

Authors	Year	Model used	Imaging device used	Dataset used		Performance metrics (%)		Input data type	Input feature-set
				**Feature set**	**Subjects used**	**Accuracy**	**Other metrics**		
Yousefi et al. [95]	2018	Unsupervised ML	SS-1000 CASIA (OCT)	420	3156	n/a	Sen: 97.7; Spe: 94.1-	IM	8
Lavric et al. [40]	2020	SVM + 24 others	SS-1000 CASIA (OCT)	8	3151	94	Sen: 89.5; Spe: 96	CP	8
Cao et al. [96]	2020	RF + 7 others	Pentacam + SS-1000 CASIA (OCT)	11	88	97	Sen: 94.0; Spe: 90.0	CP	11
Issaarti et al. [97]	2020	FNN	Pentacam	49	812	Suspect KC: 74 Other stages: 97.6 (ave)	Suspect KC: AUC: 87.0; Sen: 85.2; Spe: 70.0	CP	n/a
Hallett et al. [98]	2020	MLP + Unsupervised	Pentacam	29	124	MLP: 73 Unsupervised: 80	AUC: 89	CP	29
Aatila et al. [99]	2021	RF	SS-1000 CASIA (OCT)	446	3162	4-classes: 95	Sen: 98: Pre: 98	CP	10
Malyugin et al. [100]	2021	QDA	Pentacam	490	47,419	97	AUC: 95	CP	7
Lavric et al. [101]	2021	SVM + others	Pentacam	38	5881	n/a	AUC: 5-classes: 88	CP	3
Kamiya et al. [102]	2021	CNN	Placido topographer	n/a	179	87.78 (ave)	AUC 93.68 (ave)	IM	n/a
Shetty et al. [103]	2021	RF	Pentacam	n/a	366	91	AUC: 93; Sen: 89; Spe: 81	CP	10
Priya et al. [104]	2022	SVM	SS-1000 CASIA (OCT)	447	3164	90	Spe: 97.7; Pre: 94.1	CP	447 (all features)
Proposed system	2023	RF + 2 others	Pentacam	79	644	5-classes: 98.62 (ave)	5-classes: Sen: 98.62; Pre: 98.70; F1: 98.66; F2: 98.64	CP	3

*CNN = *convolutional neural network; *DT = *decision tree; *FNN = *feedforward neural network; *MLP = *multilayer perception neural network; *QDA = *quadratic discriminant analysis; *RF = *random forest; *SVM = *support vector machine; *AUC = *area under the curve; *F1 = *F1-score; *F2 = *F2-score; *Sen = *sensitivity (or recall); *Spe = *specificity; *Pre = *precision; *CP = *corneal parameters; *n/a = *not applicable; *ave = *average; *IM = *image (topography, tomography or Placido); *OCT = *optical coherence tomography

In contrast to the classification outcomes detailed in [101], which achieved a maximum AUC of 88% across multiple severity levels (five classes), using only three input features, our proposed classifier outperformed these results. The proposed system demonstrated high performance measured in terms of an overall accuracy of 98.62%, precision of 98.70%, sensitivity of 98.62%, F1-score of 98.66%, and F2-score of 98.64%. For studies that reported multiple models, the models with the best performance characteristics are reported in Table 6. Additionally, it is imperative to acknowledge the challenge of making direct comparisons, given the absence of a standardized grading system for categorizing KC severity across these studies [21].

### The integrated system

**Fig. 13:**
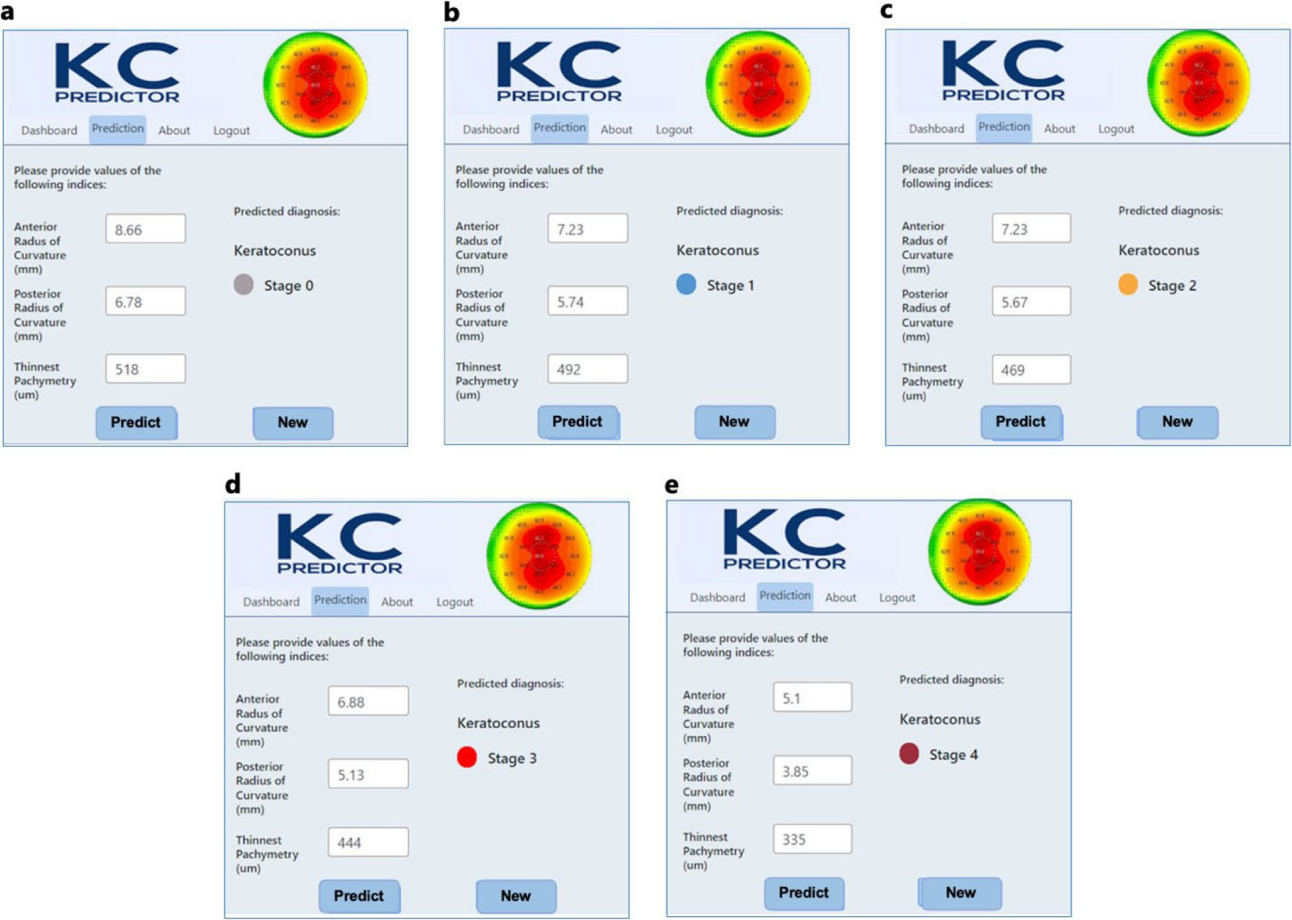
Example test results for corneas at various KC severity stages. **a** Stage 0; **b** Stage 1; **c** Stage 2; **d** Stage 3; **e** Stage 4

A fully functional decision support system for KC severity detection has been developed, successfully deployed, and tested on a web server. This system, which is collaboratively designed with ophthalmologists, is currently under additional testing to evaluate the model's generalizability. Figure 13 shows example test scenarios that represent various severity stages using new data that was not used in the training or validation test of the model. At this stage, the design of the graphical user interface remains intentionally simple to facilitate a pilot feasibility and acceptability study of the proposed system as a new diagnostic tool. These steps are considered crucial precursors to addressing challenges in implementing the system in clinical settings.

The implementation of the developed decision support system offers significant opportunities to enhance the clinical practice of KC diagnosis by:

- Facilitating the adoption of a standardized and objective diagnostic approach to severity staging by eye-care professionals, thereby reducing variability, and ensuring consistency in patient management across different practice settings.
- Increasing accessibility to KC diagnosis and severity staging across multiple eye-care facilities, irrespective of time or location.
- Providing automated analysis and interpretation of corneal curvature and pachymetry indices. This is particularly important in regions where accessing expert ophthalmologists is challenging.
- Relying on measurements obtained from a single corneal imaging device contrast with Belin's classification system, where the CDVA is a significant aspect to consider in KC severity staging.
- Assisting ophthalmologists in making informed decisions, particularly in settings where expertise in interpreting advanced diagnostic imaging is limited.

Moreover, deploying the developed application on a web server has not only enhanced its accessibility but also opened doors to new research possibilities. This includes evaluating system performance across various dimensions such as latency, stability, and security. Additionally, it enables the exploration of the feasibility and acceptability of the system as a novel KC severity screening tool in the clinical setting.

## Conclusion

The collaboration between ML experts and ophthalmologists plays a crucial role in improving clinical practice. To enhance the KC detection process, we proposed a real-world decision support system for KC staging utilising ML models and a small subset of corneal indices. The created system is a result of a close collaboration between ML experts and a team of specialist ophthalmologists. A transparent and responsible approach is adopted to feature selection, model development, validation, and deployment on a web server. This facilitates regular updates based on new data and continuous monitoring of the model’s performance that are considered fundamental aspects of the development methodology.

A reliable subset of corneal parameters that includes curvature and thinnest pachymetry indices has been identified and utilized to create a highly efficient ensemble model based on a RF classifying algorithm. The utilisation of these features has streamlined the model’s structure and considerably reduced its training time, all while preserving a high level of prediction accuracy.

The obtained findings demonstrated that the potential role of ML in KC screening is promising towards improving patient care in everyday ophthalmologic practice. To transform the developed system into a practical application, we have successfully integrated and deployed the developed model into a real-world web application server. The developed system has a promising potential as a KC severity screening tool, especially in areas lacking specialist ophthalmologists.

Future improvements for the developed system encompass multiple aspects, including:

- Evaluating the model's generalizability and interpretability.
- Updating the system post-initial deployment to align with the newly collected data and the environment.
- Exploring the implementation of advanced ensemble learning techniques to further enhance resilience and accuracy of KC detection including the severity staging.
- Exploring the feasibility of automating the transfer of corneal measurements from the Pentacam devices to our application to minimise the potential for human error and ensure more accurate and reliable data integration.
- Providing possible treatment options and referral guidelines.

These aspects, among others, constitute ongoing research endeavours of the authors.

### Supplementary Information

Supplementary Material 1.

Supplementary Material 2.

Supplementary Material 3.

## Data Availability

The datasets analysed in the current study are not publicly available due to privacy regulations set by the collaborating institutions but are available from the corresponding author upon reasonable request.

## References

[1] Bui AD, Truong A, Pasricha ND, Indaram M (2023). Keratoconus diagnosis and treatment: recent advances and future directions. Clin Ophthalmol.

[2] Goebels S, Eppig T, Wagenpfeil S, Cayless A, Seitz B, Langenbucher A (2015). Staging of keratoconus indices regarding tomography, topography, and biomechanical measurements. Am J Ophthalmol.

[3] Davidson AE, Hayes S, Hardcastle AJ, Tuft SJ (2014). The pathogenesis of keratoconus. Eye (Lond).

[4] Elubous KA, Al Bdour M, Alshammari T, Jeris I, AlRyalat SA, Roto A, et al. Environmental risk factors associated with the need for penetrating keratoplasty in patients with keratoconus. Cureus. 2021;13(7):e16506. 10.7759/cureus.16506 PMC8375353 34430120

[5] Gordon-Shaag A, Millodot M, Shneor E, Liu Y. The genetic and environmental factors for keratoconus. Biomed Res Int. 2015;2015:795738. 10.1155/2015/795738 PMC4449900 26075261

[6] Salomão MQ, Esposito A, Dupps WJ (2009). Advances in anterior segment imaging and analysis. Curr Opin Ophthalmol.

[7] Stapleton F, Alves M, Bunya VY, Jalbert I, Lekhanont K, Malet F (2017). TFOS DEWS II Epidemiology Report. Ocul Surf.

[8] Galvis V, Sherwin T, Tello A, Merayo J, Barrera R, Acera A (2015). Keratoconus: an inflammatory disorder?. Eye (Lond).

[9] Hashemi H, Heydarian S, Yekta A, Ostadimoghaddam H, Aghamirsalim M, Derakhshan A (2018). High prevalence and familial aggregation of keratoconus in an Iranian rural population: a population-based study. Ophthalmic Physiol Opt.

[10] Hashemi H, Khabazkhoob M, Yazdani N, Ostadimoghaddam H, Norouzirad R, Amanzadeh K (2014). The prevalence of keratoconus in a young population in Mashhad. Iran Ophthalmic Physiol Opt.

[11] Hashemi H, Beiranvand A, Khabazkhoob M, Asgari S, Emamian MH, Shariati M (2013). Prevalence of keratoconus in a population-based study in Shahroud. Cornea.

[12] Pearson AR, Soneji B, Sarvananthan N, Sandford-Smith JH (2000). Does ethnic origin influence the incidence or severity of keratoconus?. Eye (Lond).

[13] Ihalainen A (1986). Clinical and epidemiological features of keratoconus genetic and external factors in the pathogenesis of the disease. Acta Ophthalmol Suppl.

[14] Nielsen K, Hjortdal J, Aagaard Nohr E, Ehlers N (2007). Incidence and prevalence of keratoconus in Denmark. Acta Ophthalmol Scand.

[15] Godefrooij DA, de Wit GA, Uiterwaal CS, Imhof SM, Wisse RP (2017). Age-specific incidence and prevalence of keratoconus: a nationwide registration study. Am J Ophthalmol.

[16] Tanabe U, Fujiki K, Ogawa A, Ueda S, Kanai A (1985). Prevalence of keratoconus patients in Japan. Nippon Ganka Gakkai Zasshi.

[17] Georgiou T, Funnell C, Cassels-Brown A, O'Conor R (2004). Influence of ethnic origin on the incidence of keratoconus and associated atopic disease in Asians and white patients. Eye (Lond).

[18] Yadav SP, Yousuf B, Quantock AJ, Murphy PJ. Incidence and severity of keratoconus in Asir province. Saudi Arabia Br J Ophthalmol. 2005;89(11):1403–6. 10.1136/bjo.2005.074955 PMC1772915 16234439

[19] Ziaei H, Jafarinasab MR, Javadi MA, Karimian F, Poorsalman H, Mahdavi M (2012). Epidemiology of keratoconus in an Iranian population. Cornea.

[20] Kennedy RH, Bourne WM, Dyer JA (1986). A 48-year clinical and epidemiologic study of keratoconus. Am J Ophthalmol.

[21] Gomes JA, Tan D, Rapuano CJ, Belin MW, Ambrósio R, Guell JL (2015). Global consensus on keratoconus and ectatic diseases. Cornea.

[22] Nau AC (2008). A comparison of synergeyes versus traditional rigid gas permeable lens designs for patients with irregular corneas. Eye Contact Lens.

[23] Jeng BH, Farid M, Patel SV, Schwab IR (2016). Corneal cross-linking for keratoconus: a look at the data, the food and drug administration, and the future. Ophthalmology.

[24] O'Brart DP, Patel P, Lascaratos G, Wagh VK, Tam C, Lee J (2015). Corneal cross-linking to halt the progression of keratoconus and corneal ectasia: seven-year follow-up. Am J Ophthalmol.

[25] Kirkness CM, Ficker LA, Steele AD, Rice NS (1990). The success of penetrating keratoplasty for keratoconus. Eye (Lond).

[26] Li Y, Meisler DM, Tang M, Lu AT, Thakrar V, Reiser BJ (2008). Keratoconus diagnosis with optical coherence tomography pachymetry mapping. Ophthalmology.

[27] Esteva A, Robicquet A, Ramsundar B, Kuleshov V, DePristo M, Chou K (2019). A guide to deep learning in healthcare. Nat Med.

[28] LeCun Y, Bengio Y, Hinton G (2015). Deep learning. Nature.

[29] Yadav SP, Mahato DP, Linh NTD. Distributed artificial intelligence: a modern approach. 1st ed. CRC Press, Taylor & Francis Group; 2020.

[30] Tong Y, Lu W, Yu Y, Shen Y (2020). Application of machine learning in ophthalmic imaging modalities. Eye Vis (Lond).

[31] Feng R, Xu Z, Zheng X, Hu H, Jin X, Chen DZ, et al. KerNet: a novel deep learning approach for keratoconus and sub-clinical keratoconus detection based on raw data of the Pentacam HR system. IEEE J Biomed Health Inform. 2021;25(10):3898–910. 10.1109/JBHI.2021.3079430 33979295

[32] Ting DSW, Pasquale LR, Peng L, Campbell JP, Lee AY, Raman R (2019). Artificial intelligence and deep learning in ophthalmology. Br J Ophthalmol.

[33] Lin SR, Ladas JG, Bahadur GG, Al-Hashimi S, Pineda R (2019). A review of machine learning techniques for keratoconus detection and refractive surgery screening. Semin Ophthalmol.

[34] Klyce SD (2018). The future of keratoconus screening with artificial intelligence. Ophthalmology.

[35] Bolarín JM, Cavas F, Velázquez JS, Alió JL (2020). A machine-learning model based on morphogeometric parameters for RETICS disease classification and GUI development. Appl Sci.

[36] Velázquez-Blázquez JS, Bolarín JM, Cavas-Martínez F, Alió JL (2020). EMKLAS: a new automatic scoring system for early and mild keratoconus detection. Transl Vis Sci Technol.

[37] Kamiya K, Ayatsuka Y, Kato Y, Fujimura F, Takahashi M, Shoji N, et al. Keratoconus detection using deep learning of colour-coded maps with anterior segment optical coherence tomography: a diagnostic accuracy study. BMJ Open. 2019;9(9):e031313. 10.1136/bmjopen-2019-031313 PMC6773416 31562158

[38] Peña-García P, Sanz-Díez P, Durán-García ML (2014). Keratoconus management guidelines. Int J Keratoconus Ectatic Corneal Dis.

[39] Issarti I, Consejo A, Jiménez-García M, Hershko S, Koppen C, Rozema JJ (2019). Computer aided diagnosis for suspect keratoconus detection. Comput Biol Med.

[40] Lavric A, Popa V, Takahashi H, Yousefi S (2020). Detecting keratoconus from corneal imaging data using machine learning. IEEE Access.

[41] Cao K, Verspoor K, Sahebjada S, Baird PN (2022). Accuracy of machine learning assisted detection of keratoconus: a systematic review and meta-analysis. J Clin Med.

[42] Paleyes A, Urma R-G, Lawrence ND (2022). Challenges in deploying machine learning: a survey of case studies. ACM Comput Surv..

[43] Li Z, Wang L, Wu X, Jiang J, Qiang W, Xie H, et al. Artificial intelligence in ophthalmology: the path to the real-world clinic. Cell Rep Med. 2023;4(7):101095. 10.1016/j.xcrm.2023.101095 PMC10394169 37385253

[44] Muhsin ZJ, Qahwaji R, Ghanchi F, AI-Taee M (2024). Review of substitutive assistive tools and technologies for people with visual impairments: recent advancements and prospects. J Multimodal User Interfaces.

[45] Muhsin Z, Qahwaji R, AlRyalat S, Al Bdour M, Al-Taee M. Feature selection and detection of keratoconus using random forest and bagging. In: Yorkshire Innovation in Science and Engineering Conference (YISEC 2023). UK: Bradford; 2023. Paper no: 52. p. 1–6.

[46] de Lima Ribeiro, MF. Pentacam for keratoconus diagnosis. In: Almodin E, Nassaralla BA, Sandes J, editors. Keratoconus. Springer, Cham. 2022. p. 79–91. 10.1007/978-3-030-85361-7_9.

[47] Li J, Dai Y, Mu Z, Wang Z, Meng J, Meng T, Wang J (2024). Choice of refractive surgery types for myopia assisted by machine learning based on doctors’ surgical selection data. BMC Med Inform Decis Mak.

[48] Wang S, Minku LL, Yao X (2018). A systematic study of online class imbalance learning with concept drift. IEEE Trans Neural Netw Learn Syst.

[49] Xiao F, Slock D. Parameter estimation via expectation maximization - expectation consistent algorithm. In: 2024 IEEE International Conference on Acoustics, Speech and Signal Processing (ICASSP). Korea: Seoul; 2024. p. 9506–9510. 10.1109/ICASSP48485.2024.10447082.

[50] Lee H, Yun S (2024). Strategies for imputing missing values and removing outliers in the dataset for machine learning-based construction cost prediction. Buildings.

[51] Sandfeld S. Exploratory Data Analysis. In: Materials data science: introduction to data mining, machine learning, and data-driven predictions for materials science and engineering. Cham: Springer; 2023. p. 179–206. 10.1007/978-3-031-46565-9_9.

[52] Dastjerdy B, Saeidi A, Heidarzadeh S (2023). Review of applicable outlier detection methods to treat geomechanical data. Geotechnics.

[53] Alfian G, Syafrudin M, Yoon B, Rhee J (2019). False positive RFID detection using classification models. Appl Sci.

[54] Sheard J. Quantitative data analysis. In: Williamson K, Johanson G, editors. Research Methods: Information, Systems, and Contexts. 2nd edition. Elsevier. 2018. p. 429–52. 10.1016/B978-0-08-102220-7.00018-2.

[55] Salem BR, Solodovnikov VI. Decision support system for an early-stage keratoconus diagnosis. J Phys Conf Ser. 2019;1419(1):012023.

[56] John AK, Asimellis G (2013). Revisiting keratoconus diagnosis and progression classification based on evaluation of corneal asymmetry indices, derived from Scheimpflug imaging in keratoconic and suspect cases. Clin Ophthalmol..

[57] Luo S (2023). Synthetic minority oversampling technique based on adaptive noise optimization and fast search for local sets for random forest. Intern J Pattern Recognit Artif Intell.

[58] Ratnasari AP (2024). Performance of random oversampling, random undersampling, and SMOTE-NC methods in handling imbalanced class in classification models. International Journal of Scientific Research and Management.

[59] Elreedy D, Atiya AF (2019). A comprehensive analysis of synthetic minority oversampling technique (SMOTE) for handling class imbalance. Inf Sci.

[60] Sinjab MM (2021). Corneal tomography in clinical practice (Pentacam system): Basics and clinical interpretation.

[61] Lynch S. Python for scientific computing and artificial intelligence. 1st edition. New York: Chapman and Hall/CRC; 2023. p. 37. 10.1201/9781003285816.

[62] Kirasich K, Smith T, Sadler B (2018). Random forest vs logistic regression: binary classification for heterogeneous datasets. SMU Data Science Review.

[63] Roy A, Chakraborty S. Support vector machine in structural reliability analysis: a review. Reliab Eng Syst Saf. 2023;233:109126.

[64] Pisner DA, Schnyer DM. Chapter 6 - Support vector machine. In: Mechelli A, Vieira S, editors. Machine Learning: Methods and applications to brain disorders. Academic Press; 2020. p. 101–121. 10.1016/B978-0-12-815739-8.00006-7.

[65] Pal M (2005). Random Forest classifier for remote sensing classification. Int J Remote Sens.

[66] Misra S, Li H, He J (2020). Noninvasive fracture characterization based on the classification of sonic wave travel times. Machine learning for subsurface characterization.

[67] Lee TH, Ullah A, Wang R. Bootstrap aggregating and random forest. In: Fuleky P, editor. Macroeconomic forecasting in the era of big data. Advanced Studies in Theory and Applied Econometrics. vol.52. Springer, Cham. 2020. p. 389–429. 10.1007/978-3-030-31150-6_13.

[68] Probst P, Wright MN, Boulesteix AL. Hyperparameters and tuning strategies for random forest. Wiley Interdisciplinary Reviews: Data Mining And Knowledge Discovery. 2019;9(3):e1301.

[69] Wang X, Gong G, Li N, Qiu S (2019). Detection analysis of epileptic EEG using a novel random forest model combined with grid search optimization. Front Hum Neurosci.

[70] Bischl B, Binder M, Lang M, Pielok T, Richter J, Coors S, et al. Hyperparameter optimization: foundations, algorithms, best practices, and open challenges. Wiley Interdiscip Rev: Data Min Knowl Discov. 2023;13(2):e1484.

[71] Singh A, Akash R. Flower classifier web app using Ml & Flask web framework. In: 2022 2nd International Conference on Advance Computing and Innovative Technologies in Engineering (ICACITE). India: Greater Noida. 2022. p. 974–7. 10.1109/ICACITE53722.2022.9823577.

[72] Padhy S, Das N, Tiwari S, Arora S. AI based web app and framework for detecting emotions from human speech. In: 2022 2nd Odisha International Conference on Electrical Power Engineering, Communication and Computing Technology (ODICON). India: Bhubaneswar. 2022. p. 1–6. 10.1109/ODICON54453.2022.10010017.

[73] Lakshmanarao A, Babu MR, Krishna MB. Malicious URL detection using NLP, machine learning and FLASK. In: 2021 international conference on innovative computing, intelligent communication and smart electrical systems (ICSES). India: Chennai. 2021. p. 1–4. 10.1109/ICSES52305.2021.9633889.

[74] Hunt-Walker N. An introduction to the Flask Python web app framework: Opensource.com. 2018. Available from: https://opensource.com/article/18/4/flask. Accessed 10 June 2024.

[75] Villavicencio CN, Macrohon JJ, Inbaraj XA, Hsieh JG (2022). Development of a machine learning based web application for early diagnosis of COVID-19 based on symptoms. Diagnostics (Basel).

[76] Sculley D, Holt G, Golovin D, Davydov E, Phillips T, Ebner D (2015). Hidden technical debt in machine learning systems. Adv Neural Inf Process Syst.

[77] Quiñonero-Candela J, Masashi S, Anton S, Lawrence ND. Dataset shift in machine learning. MIT Press; 2022.

[78] Perry HD, Buxton JN, Fine BS (1980). Round and oval cones in keratoconus. Ophthalmology.

[79] Krumeich JH, Daniel J, Knülle A (1998). Live-epikeratophakia for keratoconus. J Cataract Refract Surg.

[80] Rabinowitz YS, Rasheed K (1999). KISA% index: a quantitative videokeratography algorithm embodying minimal topographic criteria for diagnosing keratoconus. J Cataract Refract Surg.

[81] Maeda N, Klyce SD, Smolek MK, Thompson HW (1994). Automated keratoconus screening with corneal topography analysis. Invest Ophthalmol Vis Sci.

[82] Alió JL, Shabayek MH (2006). Corneal higher order aberrations: a method to grade keratoconus. J Refract Surg..

[83] McMahon TT, Szczotka-Flynn L, Barr JT, Anderson RJ, Slaughter ME, Lass JH (2006). A new method for grading the severity of keratoconus: the keratoconus severity score (KSS). Cornea.

[84] Mahmoud AM, Roberts CJ, Lembach RG, Twa MD, Herderick EE, McMahon TT (2008). CLMI the cone location and magnitude index. Cornea.

[85] Li X, Yang H, Rabinowitz YS (2009). Keratoconus: classification scheme based on videokeratography and clinical signs. J Cataract Refract Surg.

[86] Sandali O, El Sanharawi M, Temstet C, Hamiche T, Galan A, Ghouali W (2013). Fourier-domain optical coherence tomography imaging in keratoconus: a corneal structural classification. Ophthalmology.

[87] Amsler M (1946). Kératocône classique et kératocône fruste; arguments unitaires. Ophthalmologica.

[88] Kamiya K, Ishii R, Shimizu K, Igarashi A (2014). Evaluation of corneal elevation, pachymetry and keratometry in keratoconic eyes with respect to the stage of Amsler-Krumeich classification. Br J Ophthalmol.

[89] Gomes JAP, Rodrigues PF, Lamazales LL (2022). Keratoconus epidemiology: a review. Saudi J Ophthalmol..

[90] Duncan JK, Belin MW, Borgstrom M (2016). Assessing progression of keratoconus: novel tomographic determinants. Eye Vis (Lond).

[91] Duncan J, Gomes J (2015). A new tomographic method of staging/classifying keratoconus: the ABCD grading system. Int J Keratoconus Ectatic Corneal Dis.

[92] Belin MW, Duncan JK (2016). Keratoconus: the ABCD grading system. Klin Monbl Augenheilkd.

[93] Belin MW, Kundu G, Shetty N, Gupta K, Mullick R, Thakur P (2020). ABCD: a new classification for keratoconus. Indian J Ophthalmol.

[94] Shetty R, Arora V, Jayadev C, Nuijts RM, Kumar M, Puttaiah NK (2014). Repeatability and agreement of three Scheimpflug-based imaging systems for measuring anterior segment parameters in keratoconus. Invest Ophthalmol Vis Sci.

[95] Yousefi S, Yousefi E, Takahashi H, Hayashi T, Tampo H, Inoda S, et al. Keratoconus severity identification using unsupervised machine learning. PLoS One. 2018;13(11):e0205998. 10.1371/journal.pone.0205998 PMC6219768 30399144

[96] Cao K, Verspoor K, Sahebjada S, Baird PN (2020). Evaluating the performance of various machine learning algorithms to detect subclinical keratoconus. Transl Vis Sci Technol.

[97] Issarti I, Consejo A, Jiménez-García M, Kreps EO, Koppen C, Rozema JJ. Logistic index for keratoconus detection and severity scoring (Logik). Comput Biol Med. 2020;122:103809. 10.1016/j.compbiomed.2020.103809 32658727

[98] Hallett N, Yi K, Dick J, Hodge C, Sutton G, Wang YG, et al. Deep learning based unsupervised and semi-supervised classification for keratoconus. In: 2020 IEEE International Joint Conference on Neural Networks (IJCNN). UK: Glasgow; 2020. p. 1–7. 10.1109/IJCNN48605.2020.9206694.

[99] Aatila M, Lachgar M, Hamid H, Kartit A (2021). Keratoconus severity classification using features selection and machine learning algorithms. Comput Math Methods Med.

[100] Malyugin B, Sakhnov S, Izmailova S, Boiko E, Pozdeyeva N, Axenova L (2021). Keratoconus diagnostic and treatment algorithms based on machine-learning methods. Diagnostics (Basel).

[101] Lavric A, Anchidin l, Valentin P, Al-Timemy AH, Alyasseri Z, Takahashi H (2021). Keratoconus severity detection from elevation, topography and pachymetry raw data using a machine learning approach. IEEE Access.

[102] Kamiya K, Ayatsuka Y, Kato Y, Shoji N, Mori Y, Miyata K. Diagnosability of keratoconus using deep learning with Placido disk-based corneal topography. Front Med (Lausanne). 2021;8:724902. 10.3389/fmed.2021.724902 PMC8520919 34671618

[103] Shetty R, Kundu G, Narasimhan R, Khamar P, Gupta K, Singh N (2021). Artificial intelligence efficiently identifies regional differences in the progression of tomographic parameters of keratoconic corneas. J Refract Surg.

[104] Priya D, Mamatha GS, Punith RM, Nagaraju G. Keratonalyse: a study of comparative analysis of supervised learning algorithms for keratoconus detection. In: 2022 International Conference on Sustainable Computing and Data Communication Systems (ICSCDS). India: Erode. 2022. p. 676–83. 10.1109/ICSCDS53736.2022.9760882.

