# Supplementary material for: Smart decision support system for keratoconus severity staging using corneal curvature and thinnest pachymetry indices
Source: Eye Vis (Lond). 2024 Jul 8;11:28. doi: 10.1186/s40662-024-00394-1 (PMC11229244; doi:10.1186/s40662-024-00394-1)
Supplement: Supplementary file 1 — Supplementary Material 1. [file 40662_2024_394_MOESM1_ESM.docx]

**Table A.1** Logistic regression

| Parameter | Value/description |
| --- | --- |
| solver | L-BFGS |
| penalty | L2 regularization (ridge regression) |
| max_iter | 10000 |
| C | 10 |
| multi_class | multinomial |

L-BFGS = limited-memory Broyden–Fletcher–Goldfarb–Shanno, a solver used to optimize the log-likelihood function of LoR
