# Supplementary material for: Smart decision support system for keratoconus severity staging using corneal curvature and thinnest pachymetry indices
Source: Eye Vis (Lond). 2024 Jul 8;11:28. doi: 10.1186/s40662-024-00394-1 (PMC11229244; doi:10.1186/s40662-024-00394-1)
Supplement: Supplementary file 2 — Supplementary Material 2. [file 40662_2024_394_MOESM2_ESM.docx]

**Table A.2** Support vector machine

| Parameter | Value/description |
| --- | --- |
| gamma | 0.0001 |
| kernel | poly |
| C | 10 |
| decision_function_shape | OvA |

OvA = one versus all, a strategy used for multi-class classification problems.
