# Supplementary material for: Smart decision support system for keratoconus severity staging using corneal curvature and thinnest pachymetry indices
Source: Eye Vis (Lond). 2024 Jul 8;11:28. doi: 10.1186/s40662-024-00394-1 (PMC11229244; doi:10.1186/s40662-024-00394-1)
Supplement: Supplementary file 3 — Supplementary Material 3. [file 40662_2024_394_MOESM3_ESM.docx]

**Table A.3** Random forest

| Parameter | Value/description |
| --- | --- |
| n_ estimators | 50 |
| Max_depth | 6 |
| min_samples_leaf | 1 |
| bootstrap | true |
| criterion | entropy |
| oob_score | true |
| random_state | 42 |
| max_features | sqrt |

sqrt = square root function
